# Evaluating causes and gestures: source-related and crossmodal features in the perception of environmental sounds

**DOI:** 10.3389/fpsyg.2025.1520209

**Published:** 2025-02-07

**Authors:** Sven-Amin Lembke

**Affiliations:** Cambridge School of Creative Industries, Anglia Ruskin University, Cambridge, United Kingdom

**Keywords:** sound gesture, environmental sound, crossmodal correspondence, pitch, loudness, source, cause

## Abstract

Communication through auditory cues often evokes associations to other sensory modalities. In film music, for instance, a descending pitch contour commonly resembles a falling motion. Such crossmodal associations to physical actions or shapes are here termed *sound gestures* and can naturally occur in environmental sounds. Little is known about how reliably listeners perceive gestures in such environmental contexts and how salient the gesture-relevant auditory feature needs to be. This article reports on an exploratory study concerning the identification of sound gestures by crossmodal matching using analogous visualizations. The study considered gesture-related factors, such as auditory salience and contour complexity, and explored whether a concurrent evaluation of features related to the environmental sound source or cause would affect gesture identification. Twenty untrained listeners evaluated sound gestures occurring in environmental sounds, e.g., pitch contour when switching a vacuum cleaner on and off, loudness contour of a ball dropping. Participants evaluated 28 environmental sounds in three variants (original, isolated gesture, hybrid) and had to identify the sound gesture among four visualized options while also inferring the underlying environmental source or cause through verbal description and rating their confidence in identifying the source/cause. Based on features describing the macro contour of gestures, participants correctly identified 81-83% of all gestures. Manipulated sounds that emphasized gesture salience yielded only slight improvements of identification accuracy compared to original environmental sounds. Participants were more confident in identifying the source/cause in sounds containing pitch gestures than those containing loudness gestures, while lexical and semantic diversity in describing underlying materials (source) and actions (cause) varied considerably. For both groups, however, measures for gesture identification and the evaluation of underlying materials and actions correlated only weakly, suggesting task independence. Overall, findings suggest that untrained listeners perceive sound gestures in environmental sounds and can reliably use them to form crossmodal associations, while also evaluating properties related to the sound source and cause. For one, the perception of environmental sounds may evoke crossmodal links, while the reliable identification of sound gestures highlights their utility to crossmodal control or search interfaces.

## 1 Introduction

Hearing environmental sounds, humans can gather a broad range of meaningful information. This *environmental* category can be defined as encompassing sounds that occur naturally and are unrelated to speech and music (Gygi et al., 2007), with others employing the comparable *everyday* label (Ballas, 1993; Guastavino, 2018; Giordano et al., 2022). Environmental sounds can be evaluated and distinguished from speech and musical sounds within fractions of a second (Ogg et al., 2017), and it is argued that their main utility is to help humans explain the causality of environmental events surrounding them (Giordano et al., 2022).

Through listening to environmental sounds, humans can infer what events occur, what underlying sources cause them, how and where events take place, when they occur, and how they evolve over time (see Guastavino, 2018; Giordano et al., 2022, for recent reviews). Among this range of semantic categories and labels, the most prevalent concern the sources or objects (what) and actions (how) that underlie environmental sounds (Guastavino, 2018; Giordano et al., 2022).

The relevance of object and action as two common, basic categories has been confirmed across a range of perceptual studies focusing on the factors underlying the identification of environmental sounds (e.g., Ballas, 1993; Lemaitre et al., 2010; Houix et al., 2012). Studies have employed verbal description in pairs of nouns and verbs for objects and actions, respectively (Ballas, 1993; Lemaitre et al., 2010), while others have described objects also in terms underlying materials (Gaver, 1993; Giordano and McAdams, 2006; Houix et al., 2012; Lemaitre and Heller, 2012; Hjortkjær and McAdams, 2016; Lemaitre et al., 2018). Furthermore, Ballas (1993) established that the accuracy for correct identifications and the associated response time depends on factors such as familiarity, ecological frequency, and the identifiability (rated confidence in knowing the source/cause).

Lemaitre et al. (2010) have shown that the level of listening expertise predicts different tendencies for perceptually evaluating environmental sounds. Lower expertise favors the evaluation of causal features (e.g., object, action), whereas listeners with greater expertise tend to evaluate acoustic features, in other words, related to sound qualities such as pitch or loudness. In addition, the same study has found a weaker general tendency for less confidence regarding the source/cause, or conversely greater uncertainty, to favor the evaluation of acoustic over causal features. In summary, the results by Lemaitre et al. (2010) suggest that sound source and sound qualities form polar opposites regarding which of the features governs listeners' perceptual evaluation.

From a theoretical and phenomenological perspective, the perceptual distinction between sound sources and qualities has been argued to concern different modes of listening. Whereas Gaver (1993) distinguishes between *everyday* and *musical* listening as applying to source/cause and qualitative properties, respectively, this distinction parallels the one between *causal* (Chion, 1994) and *reduced* (Chion, 1994; Schaeffer, 2017) listening modes. Theorists employing these distinctions agree with the former, ecologically more relevant mode acting by default, with Smalley (1997) employing the term *source bonding* to illustrate humans' natural tendency to attend to the identity of sources and underlying causes when they are readily apparent.

In line with the aforementioned opposition between sound sources and qualities, Smalley (1997) assumes that source bonding counteracts listeners' ability to attend to sound qualities. Smalley approaches this argument as a composer of electroacoustic music, a genre in which any type of sound (e.g., speech, environmental, synthesized) can be used and experienced musically if relevant sound qualities, such as pitch, loudness or timbre, are sufficiently salient. The temporal shaping of such sound qualities is argued to give rise to the perception of sound *shapes* or *gestures* that draw on extrasonic, crossmodal references (see Lembke, 2023, for a review).

Whereas the perception of sound-to-shape analogies has been studied in various contexts (e.g., Adeli et al., 2014; Thoret et al., 2014; Lembke, 2018), the perception of sound gestures applies to the wider study of crossmodal correspondences (see Spence, 2011; Deroy and Spence, 2016, for reviews) and how they underpin mappings between sound qualities and visual or spatial dimensions (see Lembke, 2023, for a review). In a two-dimensional visual frame, for instance, listeners intuitively understand pitch to relate to the vertical dimension (Spence, 2011; Athanasopoulos and Moran, 2013; Küssner and Leech-Wilkinson, 2014; Lacey et al., 2020; Lembke, 2023), while they can understand the horizontal dimension to represent time (Athanasopoulos and Moran, 2013; Küssner and Leech-Wilkinson, 2014; Lacey et al., 2020; Lembke, 2023). When evaluated in isolation, also loudness can be matched to the vertical dimension (Eitan et al., 2008; Küssner and Leech-Wilkinson, 2014; Bruzzi et al., 2017; Lembke, 2023). Such two-dimensional visual analogues that depict the time course of auditory qualities already find wide application, such as in western musical notation (for pitch), sound waveforms (for amplitude/loudness), visualization of speech prosody (e.g., Hermes, 1998).

By evaluating two-dimensional visual interfaces that represent pitch or loudness, viewers can identify meaningful categories, such as a *rise* or *fall* of pitch or a sudden *impact* as opposed to a gradual *decay* in loudness, all of which can infer extrasonic, actual or metaphorical spatiokinetic processes. Apart from these semantic categories or units, Lembke (2023), employing a crossmodal-matching task, has shown that both pitch- and loudness-based sound gestures can be reliably distinguished in their shape. For instance, listeners can describe a pitch *rise* also in terms of different degrees of curvature. Taken together, sound gestures can therefore be perceived and described at two morphological levels: the category (general orientation) and shape (curvature over time).

Returning to the notion that environmental sounds can convey how events occur over time (Guastavino, 2018; Giordano et al., 2022), there are many situations in which these sounds contain audible sound gestures. Common examples are pitch glides that occur during the operation of machines or dynamic trajectories of sound sources involving tonal components or filtered noise, with these cues remaining largely unexplored (e.g., Lemaitre et al., 2017, investigating a few exemplars). It therefore remains unclear to what extent sound gestures occurring in environmental sounds are perceived and how perception may depend on their auditory salience and complexity. Furthermore, given the presumed opposition between sound source/cause and sound qualities (see Smalley, 1997; Lemaitre et al., 2010), studying gesture perception alongside concurrent perception of source/cause properties merits special attention.

This article reports on an exploratory study concerning the identification of sound gestures that naturally occur in environmental sounds. By measuring gesture identification through crossmodal interfaces, the investigation explored gesture-related factors such as auditory salience, contour and acoustic complexity, while engaging participants with the concurrent evaluation of sound properties related to the source or cause and studying their potential influence on gesture identification. The following sections first specify the perceptual experiment investigating these aims, followed by the presentation and discussion of results.

## 2 Materials and methods

### 2.1 Stimuli

Sound stimuli involved gestures expressed through either pitch or loudness variation, which naturally occurred in a set of environmental sounds. Each sound gesture was presented in three conditions or sound types: (1) the *original* environmental sound, (2) a *noise*-based isolated gesture, and (3) a *hybrid* of the two. Whereas the *original* sounds exhibited acoustical cues that conveyed both the gesture and the sounds' source or cause, the noise-based sounds were designed to obscure cues for source/cause identification.

All tested sound stimuli were produced in digital PCM format at 44.1 kHz sampling rate and 24-bit dynamic resolution. Approximate matching in loudness among all sounds was achieved by equalization of root-mean-square (RMS) amplitude.

#### 2.1.1 Original environmental sounds

Environmental sounds that exhibited pitch or loudness gestures concerned three origins. Some sounds were downloaded from the *Freesound* webpage,^1^ while others concerned previously recorded material made available to the author by an academic colleague. In addition, more than half of the sounds were recorded by the author in domestic or studio settings and involved manual handling of household items and common materials. All sources yielded a total of 30 and 36 sounds conveying pitch and loudness gestures, respectively.

For the main experiment, these two sets were reduced down to 14 sounds for each gestural parameter to limit the experiment's scope and duration. The sub-selection was informed by results from a pilot experiment with five untrained listeners, who were asked to choose the correct gesture out of four options after hearing an isolated, *noise*-based gesture; this employed the same crossmodal-matching task described in Section 2.3. The 14 sounds for each parameter were sampled across the range of 60–100% of correct identification. Gestures exhibiting identification rates below that range were not included because 1) they were deemed even less likely to be identified when occurring in the *original* environmental sounds and 2) they were less likely to yield statistical differences from 25% chance level for the anticipated sample size of 20 participants.

Tables 1, 2 list the 2 × 14 selected sounds. Numerals at the end of a sound title denote its serial position among excerpts from a longer recording of similar sounds. Excluded sounds concerned variants of same sound sources/causes (e.g., Rasp 6, Dominos 18) and other sources such as chalk screech, slide whistle, firework rocket, grains in a glass container, and violin or timpani glissandi. For each gesture parameter, six additional sounds were selected as stimuli for the practice trials, e.g., door creak, chalk screech, door stopper, window wind, balls falling, metal dig; they did not feature in the main experiment.

**Table 1:** Fourteen environmental sounds containing pitch-based gestures.

**Sound source**	**Additional description**	**Duration in s**	**Origin**
Filling water carafe	Begins empty	7.11	Self-recorded
Hair dryer 1	Switched on and off, “high” setting	2.36	Self-recorded
Hair dryer 4	Switched on and off, “low” setting	3.13	Self-recorded
Kitchen mixer	Switched on and off	4.66	Self-recorded
Vacuum cleaner	Switched on and off	7.86	Self-recorded
Car revving	Stationary, revving three times	8.12	Freesound ID 327416
Car accelerating	Driving past on road	10.41	Freesound ID 341608
Plane flying overhead	Approaching and receding	13.73	Freesound ID 392486
Door creak 1	Swinging open/close	4.17	Freesound ID 458457
Door creak 3	Brief hinge action	1.97	Freesound ID 458458
Door creak 2	Swinging open/close	5.96	Freesound ID 458459
Window wind 1	Draught through a gap	9.05	Freesound ID 9097
Window wind 2	Draught through a gap	10.42	Freesound ID 9097
Rasp 2	Single swipe along rasp	3.33	Recorded by colleague

**Table 2:** Fourteen environmental sounds containing loudness-based gestures.

**Sound source**	**Additional description**	**Duration in s**	**Origin**
Falling coins 4	On wood floor	1.91	Self-recorded
Falling coins 7	On wood floor	2.09	Self-recorded
Oven grill 1	Strummed across	3.56	Self-recorded
Oven grill 3	Strummed across	3.13	Self-recorded
Drawer	Sliding shut	2.00	Recorded by colleague
Metal dig	On gravel/soil	1.31	Recorded by colleague
Rasp 5	Aperiodic swipe along rasp	1.32	Recorded by colleague
Balls falling 4	Two sync. tennis balls, on board	2.82	Self-recorded
Balls falling 6	Two async. tennis balls, on board	2.63	Self-recorded
Balls falling 18	One squash ball, on board	1.99	Self-recorded
Dominos 2	Wood pieces toppling	2.10	Self-recorded
Dominos 6	Wood pieces toppling	2.23	Self-recorded
Pen cap	Plastic scribbling on metal	1.72	Self-recorded
Felt marker	Scribbling, sticking and slipping on whiteboard	2.87	Self-recorded

#### 2.1.2 Noise-based, derivative sounds

Noise-based sounds were synthesized reductions of the original sounds that isolated and exposed the gestures by attempting to remove identifying cues for the sound source or cause. Synthesis relied on extracting the relevant audio feature that conveyed the gestural parameter pitch or loudness.

##### 2.1.2.1 Pitch gestures

In the original sounds, the auditory gesture was conveyed by a tonal quality varying over time, which related to the parameters pitch or timbral brightness; both can be considered equivalent in the context of gestures or contours (McDermott et al., 2008). The underlying acoustic cue concerned the relevant tonal component in terms of its frequency and time course. Extracting and rendering a continuous frequency from the original sound involved two stages: 1) manual treatment of the original sound in Audiosculpt software (IRCAM, 2016) and 2) automated extraction and further treatment in MATLAB (MathWorks, 2018).

In Audiosculpt, using an editable spectrogram interface, the frequency component highest in amplitude and its time course was identified. Its trajectory was isolated by attenuating all surrounding spectral regions by the maximum possible gain (-116 dB). These treatments were exported as audio files, and it was ascertained through hearing that the result conveyed the relevant pitch gesture in an isolated, exposed manner.

In MATLAB, feature extraction of the pre-treated sound was based on a cochleagram representation generated by a gammatone filterbank (Hohmann, 2002), configured with 78 bands, a 100 Hz lower limit, and a frequency resolution of two bands per equivalent-rectangular bandwidth (ERB, Moore and Glasberg, 1983). Next, a moving-average filter (Hann window, length: 7 bands) applied spectral smoothing to the cochleagram, after which the frequency trajectory was derived by evaluating the spectral maxima across time frames.

These trajectories still required further treatment in MATLAB, because at low signal amplitudes they could be contaminated by unrelated signal noise. To filter out these noise artifacts, trajectory values were gated/removed when their corresponding amplitudes fell below a pre-defined threshold, which was determined heuristically for each sound (range: -24 to -60 dB relative to maximum). Gaps resulting from the gating were inter- or extrapolated using the nearest-neighbor method. Subsequent smoothing over time using a low-pass filter (Butterworth, 4th order, cutoff frequency: 5 Hz) yielded the final, continuous frequency trajectories.

The noise-based stimuli were based on bandpass-filtered pink noise. The pitch gestures were articulated through time variation of the filter's centre frequency, based on the gesture's frequency trajectory. The band-pass filter (Butterworth, 2nd order) exhibited a constant bandwidth of *Q* = 23.1 (one-sixteenth octave). The isolated gesture's temporal amplitude envelope was imposed on the resulting filtered noise. For these noise-based gestures, the initial sound-level matching based on RMS amplitude required some heuristic adjustments to several sounds to achieve more similar loudnesses, e.g., -9 dB attenuation of rasp, +3 dB amplification of car, plane, and wind sounds.

##### 2.1.2.2 Loudness gestures

In the original sounds, the auditory gesture was conveyed by the temporal variability in loudness, which at the same time conveyed rhythmic traits. The acoustic cue underlying the loudness variation concerned the temporal amplitude envelope, which had to be extracted from the original sounds.

Using MATLAB, the original sounds' amplitude envelope was extracted by low-pass-filtering the original signal (Butterworth, 4th order, cutoff frequency: 125 Hz), comparable to approaches by Shafiro (2008) or Agus et al. (2012). The noise-based loudness gestures were synthesized by imposing the extracted amplitude envelope onto a signal containing bandpass filtered pink noise. The same filter structure was used as for the pitch gestures, only that for loudness gestures the center frequency remained fixed at 1 kHz.

#### 2.1.3 Hybrid sounds

Hybrid sounds for both pitch and loudness gestures entailed a mixture of the *original* and the *noise-based* sounds, with both matched in root-mean-square (RMS) amplitude. Given that the filter structures underlying the noise-based sounds introduced delays, negative system delays were applied to the noise-based sounds to ensure their time alignment with the original sounds.

#### 2.1.4 Visual analogues for sound gestures

To measure the identification of auditory gestures, we employed a crossmodal matching task that used two-dimensional visual analogues. As will be elaborated on in Section 2.3, four visual response options were needed for a gesture, while only one reflected the correct visual analogue.

The visual analogues depicted gestures in black on a white background and resembled hand-drawn sketches of a felt marker on a whiteboard. A computational approach generated these based on the two physical dimensions underlying the gesture. For both parameters, the horizontal dimension (x-axis) represented linear time progressing from left to right, whereas the vertical dimension (y-axis) corresponded to either pitch (bottom: low, top: high) or loudness (bottom: soft, top: loud). As individual data points of the gesture trajectories occupied only single pixels and were less discernible, they were enlarged by two-dimensional convolution with Tukey windows (length: 2–4% of total pixels, cosine-taper ratio: 0.5 for loudness, 0.9 for pitch). The visualization was scaled to fit a sound in question, based on its duration and frequency/amplitude range, and included a white border.

##### 2.1.4.1 Pitch gestures

These visual analogues relied on the gestures' frequency trajectories (see Section 2.1.2). The vertical dimension represented frequency along ERB rate, as this psychoacoustic scale has been deemed the most suitable transfer function to map between auditory pitch and visual shape (Hermes, 1998; Lembke, 2023).

Table 3 displays the visual analogues for all 14 pitch gestures (in column I) and their three response alternatives (in columns II–IV), which were derived from the correct visual analogue (labelled I) by flipping horizontally and/or vertically, that is, corresponding to time reversal (II), frequency inversion (III), and both reversal and inversion (IV).

**Table 3:** Visual analogues for the 14 pitch gestures (rows) across the response options I–IV (columns).

**Sound source**	**I**	**II**	**III**	**IV**	**Macro contour**
Filling water carafe	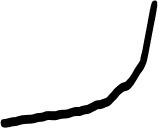	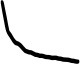	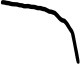	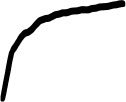	Rise
Hair dryer 1	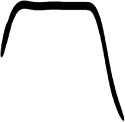	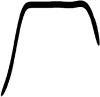	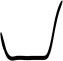	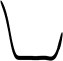	Rise, plateau, fall
Hair dryer 4	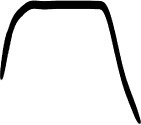	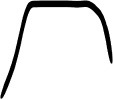	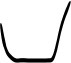	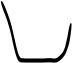	Rise, plateau, fall
Kitchen mixer	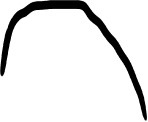	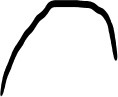	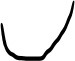	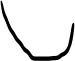	Rise, plateau, fall
Vacuum cleaner	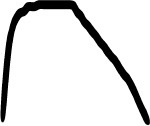	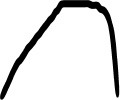	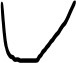	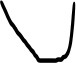	Rise, plateau, fall
Car revving	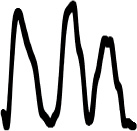	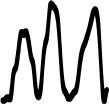	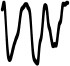	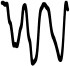	3 × Rise, fall
Car accelerating	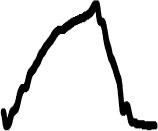	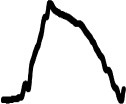	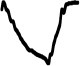	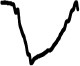	Rise, fall
Plane flying overhead	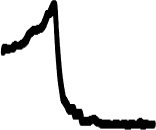	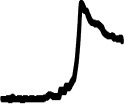	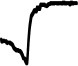	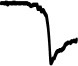	Rise, fall
Door creak 1	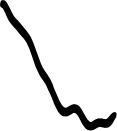	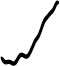	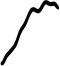	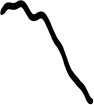	Fall
Door creak 3	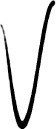	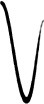	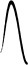	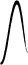	Fall, rise
Door creak 2	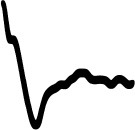	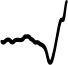	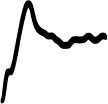	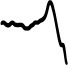	Fall/rise, rise/fall, plateau
Window wind 1	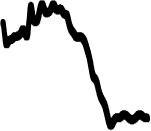	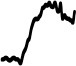	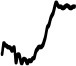	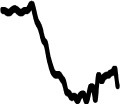	Fall
Window wind 2	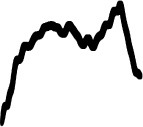	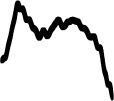	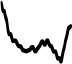	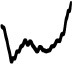	Rise, plateau, fall
Rasp 2	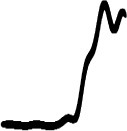	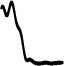	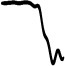	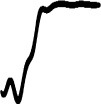	Rise

The correct gesture (I) is visualized largest, whereas the next largest option matches option I in macro contour (see description in rightmost column).

Notably, one of the alternatives (visualized second largest) matched the correct option (visualized largest) in terms of macro contour: e.g., as the contour *rise, plateau, fall* is symmetric over time, its reversal yields the same macro contour. Visible differences in fine-grained contour, reflecting nuances in gestural shape, were evident regardless.

##### 2.1.4.2 Loudness gestures

These visual analogues relied on loudness gestures' temporal amplitude envelope. They resembled rectified waveforms (e.g., employed on *Soundcloud* website,^2^) although negative signal polarities were in fact accounted for in the absolute amplitude envelope. The vertical dimension represented linear amplitude as opposed to a logarithmic sound-level transformation, as crossmodal matching of loudness gestures more closely approximates a linear function (Lembke, 2023). To emphasize signal content above the ambient noise, the visualizations excluded amplitudes below the 10th percentile.

Table 4 displays the 14 loudness gestures visual analogues (Column I) and their three response alternatives (columns II–IV). Only one (II) of the three was derived from the correct visual analogue (I) and corresponded to its time reversal (horizontal flip). As an inversion along amplitude makes little sense, the remaining two alternatives were drawn from a separate environmental sound that was comparable in terms of source or cause and/or gesture. The additional sound in its original time orientation served as the third option, whereas the fourth alternative involved its time reversal. One of the three alternative response options (visualized second largest) again exhibited a similarity in macro contour to the correct response option (visualized largest). Here, macro contour considered prominent amplitude-envelope characteristics such as impulsive, iterative, decaying morphologies (comparable to Peeters and Deruty, 2010; Schaeffer, 2017). In 11 cases, this concerned the additional sound (III). Visible differences in fine-grained contour are apparent nonetheless, e.g., brief pauses, iterative or rhythmic differences.

**Table 4:** Visual analogues for the 14 loudness-based gestures across the response options I–IV.

**Sound source**	**I**	**II**	**III**	**IV**	**Macro contour**
Falling coins 4	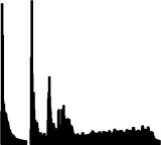	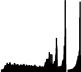	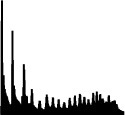	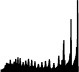	Impulses, stable, decay
Falling coins 7	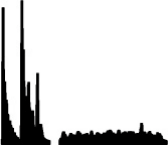	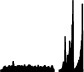	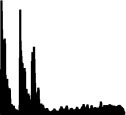	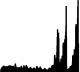	Impulses, stable, decay
Oven grill 1	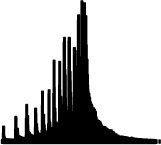	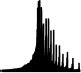	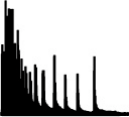	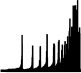	Iteration, decay
Oven grill 3	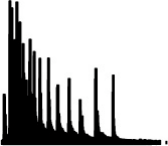	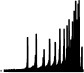	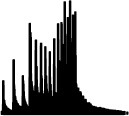	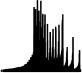	Iteration, decay
Drawer	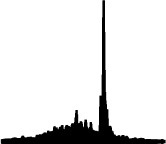	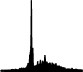	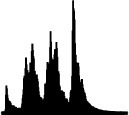	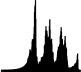	Build up, impulse, decay
Metal dig	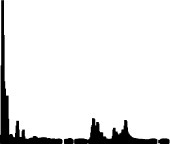	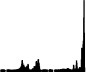	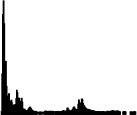	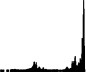	Impulse, stable, decay
Rasp 5	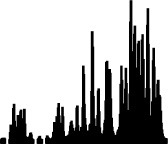	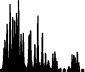	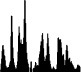	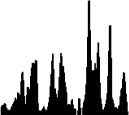	Iterative build up
Balls falling 4	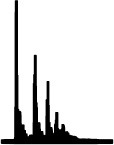	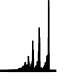	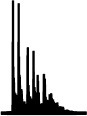	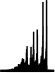	Impulses, decay
Balls falling 6	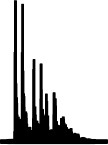	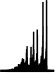	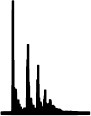	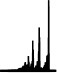	Impulses, decay
Balls falling 18	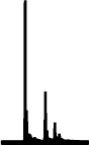	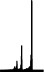	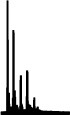	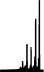	Impulses, decay
Dominos 2	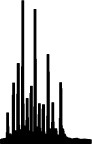	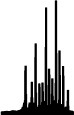	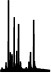	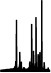	Iteration (without interruption)
Dominos 6	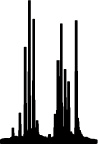	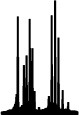	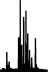	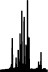	Iteration (with interruption)
Pen cap	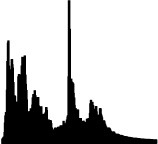	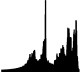	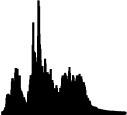	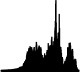	Iteration, impulse(s), decay
Felt marker	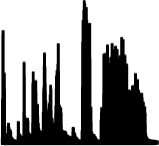	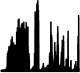	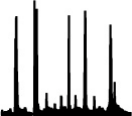	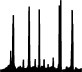	Iteration, decay

The correct gesture (I) is visualized largest, whereas the next largest option matches option I in macro contour (see description in rightmost column).

### 2.2 Participants

The study sought to assess to what extent sound gestures occurring in environmental sounds could already be perceived and identified by normal, untrained listeners. Recruitment therefore considered the general population, involving the wider community of De Montfort University, Leicester, United Kingdom, through cross-departmental advertisements that targeted all age groups and academic backgrounds. Twenty participants completed the experiment (age range: 18–65, gender: 12 female, 8 male), including one who reported ongoing hearing issues (impaired audibility threshold). Participation in the experiment involved informed consent, and the procedure had received prior approval by the Research Ethics Committee of De Montfort University. Participants were offered remuneration for their involvement.

Ten participants classified themselves as non-musicians, nine as amateur musicians, and one as professional musician. In terms of years of formal training in music or audio-related disciplines, 75% of participants (3rd quartile) indicated 0 years across the categories ear training, harmony, composition, music analysis, music history, audio diffusion or sonification, audio synthesis and processing. For the same distributional statistic, formal training amounted to no more than 1 year for audio editing, mixing, recording and less than 4 years for musical-instrument performance. Overall, this suggests that a large majority of participants had a relatively low degree of listening expertise.

### 2.3 Procedure

**Figure 1:**
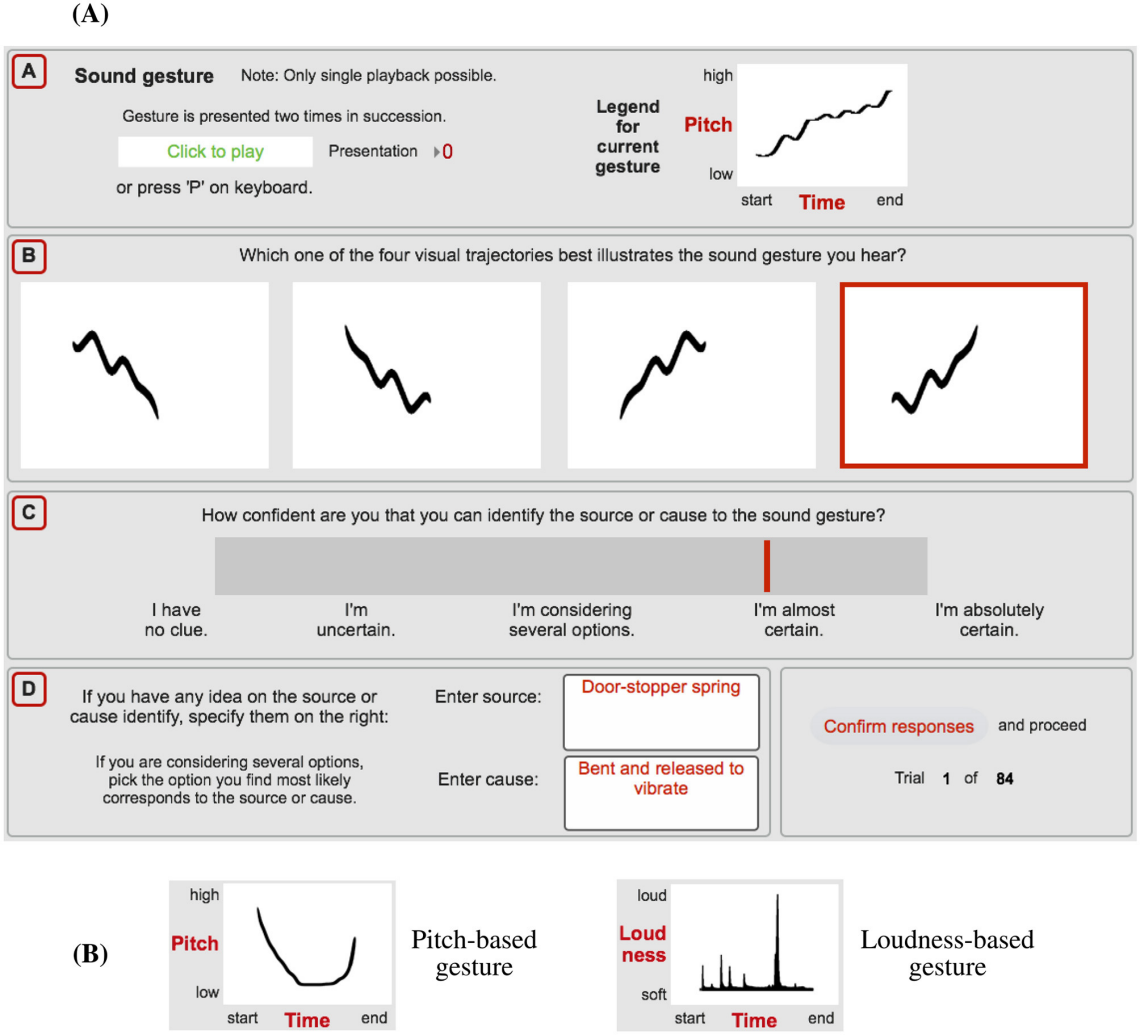
**(A)** GUI used for a single experimental trial (e.g., pitch gesture occurring in doorstopper spring), involving the four tasks listening [A], identifying the corresponding visual-analogue gesture [B], rating the source/cause confidence [C], and describing the material (source) and action (cause) [D]. **(B)** Examples of visual guidance provided to participants on how to interpret visual analogues.

The experiment tested participants' ability to identify pitch/loudness gestures occurring in sounds along with their ability to identify the underlying source and cause. In a single experimental trial, participants engaged with four tasks, depicted as the labelled sections A–D in Figure 1A. The graphical user interface (GUI) and experimental environment was implemented in Max/MSP software (Cycling '74, 2018). Task A required participants to listen to the sound in question, which was presented twice in succession. For Task B, using crossmodal matching, participants had to identify and select the auditory gesture they heard among the four visual analogues presented (in Figure 1A, the selection framed in red). The four response options to a given gesture are shown in Tables 3, 4; their spatial arrangement was randomized for each trial. Task C involved participants rating how confident they were in identifying the sound's source or cause. The continuous rating scale included five verbal labels, spaced equally apart along its length; the descriptions were equivalent in meaning to a similar approach employed by Lemaitre et al. (2010). Task D expanded the consideration of the source/cause by asking participants to describe in words the source material/object and causal action (verb), if they were sufficiently confident to determine any. Figure 1A provides example responses for a trial involving the sound of a door-stopper spring that conveys an undulating, upward-oriented pitch gesture.

In the main experiment, the two sets of sounds involving 14 pitch and 14 loudness gestures (see Tables 1, 2) were presented in separate, alternating blocks, whereas the sounds' order within blocks was randomized. The block order was counter-balanced across participants, based on which odd-numbered participants began with pitch-gesture blocks while even-numbered participants began with loudness-gesture blocks.

As established in Section 2.1, the experiment considered three types of sounds, namely *original, noise-based*, and *hybrid*; audio files featuring all sounds are available in the Supplementary material to this article. The distinction between sound types allowed the identification of differences in identification performance between when gestures were occurring in environmental sounds or emphasized through noise-based sounds. These sound types were grouped into higher-level blocks that encapsulated the aforementioned pitch- and loudness-gesture blocks. Based on their function, the order of the sound-type blocks was intentionally fixed to (1) *original*, (2) *noise-based*, and (3) *original* or *hybrid*. Comparisons between the first two blocks and the third block would allow assessing the role of repeated presentation and prior familiarity.

The *original* sounds were presented first to rule out the anticipated advantage of prior exposure to isolated gestures. The *noise-based* sounds followed in the next block to present gestures in an isolated, exposed state. The final block entailed either a repetition of the *original* or the presentation of the *hybrid* sound. Compared to the former, the latter was expected to emphasize gestural features. In total, participants underwent 84 experimental trials, composed of 3 sound types × (14 pitch gest. + 14 loudness gest.). The distinction between *original* and *hybrid* in the final block was counterbalanced across participants and gesture stimuli: e.g., participant 1 was presented 7 *original* + 7 *hybrid* pitch/loudness gestures, while participant 2 was presented the 7+7 complement. As a result, Block 3 conditions for *original* and *hybrid* were each evaluated by only half the number of participants.

Given the important role of the crossmodal matching between auditory gestures and their visual analogues, participants received both written and visual guidance on how to interpret the visual analogues. This included the two examples depicted in Figure 1B that used labels to illustrate the visual layout of time and gesture-parameter dimensions. Before the main experiment, participants conducted 12 practice trials under supervision of the experimenter, which allowed them to clarify questions. The practice trials entailed six different pitch gestures, half presented as *original* and half as *noise-based* sounds, followed by six loudness gestures, similarly partitioned across the two sound types; as stated in Section 2.1.1, the practice stimuli were not used in the main experiment.

The experiment took place in a relatively sound-absorbent and -isolated booth (volume: 15.4 m^3^, reverberation time: *T*_30_ = 0.45 s). The booth was primarily used as a 5.1-surround sound editing and mixing suite and, apart from the loudspeakers, was equipped with two computer flat screens, mouse, and keyboard, standing on a table situated in the center of the room. The sound stimuli were mainly presented via the center speaker of the 5.1 setup, a Genelec *8040A* active loudspeaker, while frequencies below 85 Hz were reproduced by a Genelec *7070A* active subwoofer; the latter was located on the floor, adjoining the back wall. Participants faced the center loudspeaker on-axis at a distance of about 1.2 m. An RME *Fireface UFX* audio interface processed the digital-to-analog conversion using the original sample rate and dynamic resolution (see Stimuli).

## 3 Results

For each sound, the behavioral data entailed a binary outcome on sound-gesture identification (correct/incorrect), a continuous rating regarding participants' confidence in identifying the sound source/cause, and two free-text responses with verbal descriptions of the *material* (related to the source) and *action* (related to the cause). This range of data representations will first be discussed separately for gesture identification (Section 3.1) and for sound source/cause confidence and verbal description (Section 3.2) before evaluating the relationship between gesture and source/cause identification (Section 3.3).

### 3.1 Sound-gesture identification

The analysis of binary correct/incorrect responses for sound-gesture identification concerns their aggregation across participants and other experimental conditions, which is expressed as the proportion of correct responses based on a relevant sample size *N*. The proportions are accompanied by estimates of 95% confidence intervals, while pair-wise comparisons of proportions employ the χ^2^ test of independence.

For both pitch and loudness gestures, the task of matching the heard gesture to the correct visual analogue involved four response options. Among the four options, only one visual analogue matched the sound gesture (in its correct orientation), which will be denoted as *strict* classification. Correct identification therefore concerned 25% chance level and accounted for listeners evaluating gestures based on relatively fine-grained characteristics.

Alternatively, listeners could have relied on less detailed distinctions by evaluating only features related to gestures' macro contour. Among the four response options, two response options could be deemed equivalent in macro contour, which will be denoted as *relaxed* classification and concerned 50% chance level. As shown in Table 3 for the pitch gesture occurring in *Hair dryer 1* (row 2), both response options I and IV exhibit the macro contour of *rise, plateau, fall*. Likewise, as shown in Table 4 for the loudness gesture occurring in the sound *Oven grill 1* (row 3), both options I and III exhibit the contour *iteration, decay* and would thus be considered correct based on *relaxed* classification.

**Figure 2:**
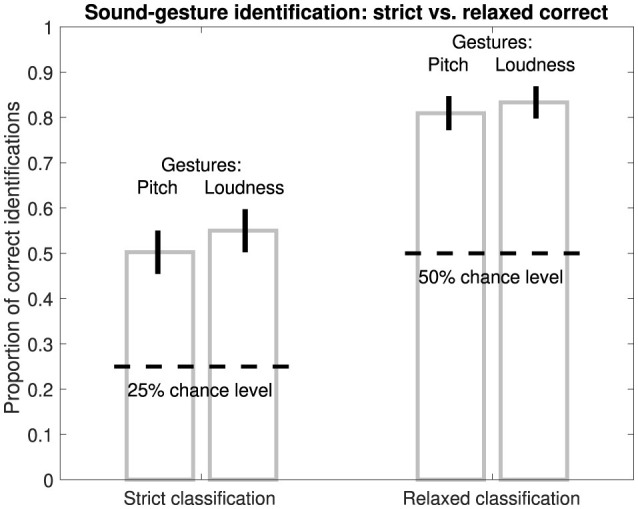
Proportion of correct identifications of pitch/loudness gestures in *original* sounds based on *strict* (1 of 4) or *relaxed* (2 of 4) classification. Latter is based on equivalence of two visual analogues in macro contour; see Tables 3, 4. Error bars represent 95% confidence intervals (*N* = 420).

Figure 2 compares correct identification of *original* environmental sounds following *strict* and *relaxed* classification across all pitch and loudness gestures (left and right bars, respectively). Whereas for *strict* classification identification accuracy amounted to around 50–55%, for relaxed classification, participants were able to correctly identify gestures in 81–83% of cases. For both classification approaches, identification performance was well above chance level. As identification performance in absolute terms was higher and more meaningful for the *relaxed* classification, all subsequent evaluations and analyses of gesture identification report on proportion correct for this identification measure.

**Figure 3:**
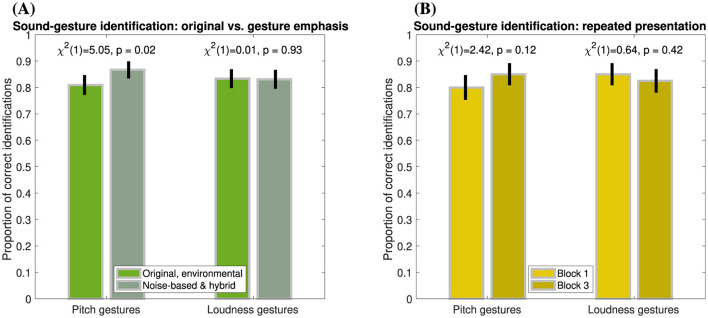
Proportion of correct identifications of pitch and loudness gestures (left and right pairs of bars, respectively) based on *relaxed* classification. Error bars represent 95% confidence intervals. **(A)** Gesture emphasis (*N* = 420), *original* vs. *noise-based* sounds (incl. *hybrid*). **(B)** Repeated presentation (*N* = 280), Block 1 (*original* sounds) vs. Block 3 (*original* and *hybrid* sounds).

The previous data concerned gesture-identification performance in *original* environmental sounds. Since greater emphasis on gestural features may facilitate gesture identification, however, the following analysis compares identification performance between *original* sounds, in which gestural features occur alongside acoustical cues conveying sound source/cause identity, and the two sound types that emphasize gestural features by isolation (*noise-based*) or acoustical emphasis (*hybrid*). Figure 3A distinguishes gesture-identification performance between these two groups. For pitch gestures (left pair of bars), gesture emphasis yielded a slight improvement in identification by 6% (χ^2^(1)=5.1, *p* = 0.02), while gestures occurring in *original* sounds were still correctly identified in 81% of cases. For loudness gestures (right pair of bars), gesture identification of around 83% did not vary across sound types (χ^2^(1) < 0.1, *p* = 0.93).

Another group-based comparison concerns the possible role of repeated presentation of the same gestures, where the effect of prior familiarity could be expected to improve gesture-identification performance. As shown in Figure 3B, for both pitch and loudness gestures (left and right panels, respectively) occurring in *original* and *hybrid* sounds, no clear, consistent differences in identification accuracy were apparent between Block 1 and Block 3 (pitch: χ^2^(1) = 2.4, *p* =.12, loudness: χ^2^(1) = 0.6, *p* = 0.42).

**Figure 4:**
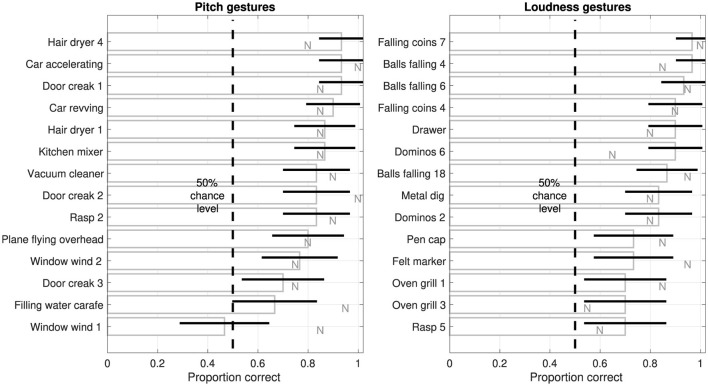
Proportion of correct identifications of individual gestures in *original* sounds, ordered from highest to lowest, for all 14 pitch and 14 loudness gestures (left and right, respectively) based on *relaxed* classification. Error bars represent 95% confidence intervals (*N* = 30). For comparison, letters N represent the corresponding identification accuracy for *noise-based* sounds.

Figure 4 presents gesture-identification performance across the individual 14 pitch and 14 loudness gestures occurring in *original* environmental sounds, ordered from highest to lowest accuracy. Although identification accuracy varied across individual gestures, performance remained above chance level for almost all gestures. For pitch gestures (left panel), a group of sounds entailing domestic electric appliances, door creaks, and car sounds achieve gesture-identification accuracies above 83%, whereas accuracy for the remaining sounds spans a wider range. Accuracy for the sound *Filling water carafe* was only marginally above chance level, whereas only a single sound, *Window wind 1*, performed at chance level. Notably, *noise-based* reductions (see letters N) of the same two environmental sounds achieved considerably higher accuracies (*carafe*: χ^2^(1) = 5.6, *p* = 0.02, *wind*: χ^2^(1) = 7.5, *p* = 0.01), representing the only sounds for which such differences attained statistical significance. For loudness gestures (right panel), sounds involving falling coins or balls mostly achieved accuracies of 90% and above, whereas the range of remaining sounds extended to the minimum accuracy of 70%. Only the gesture occurring in the *Dominos 6* sound exhibited a statistically significant difference to its *noise-based* version (see letter N, χ^2^(1) = 4.7, *p* = 0.03), with identification accuracy being markedly higher for the *original* environmental sound.

### 3.2 Sound source/cause identification and description

The second aspect of sound perception studied concerned the identity of the source and/or cause, in which context mainly the environmental *original* sound type was relevant. Listeners' identification ability was evaluated in two ways: (1) a continuous rating of participants' confidence in identifying the sound source/cause, (2) verbal descriptions for the *material* and *action* underlying the sounds, representing the source and cause, respectively. Whereas the confidence ratings allowed direct quantification, the verbal descriptions were qualitative in nature, from which a quantitative measure for causal uncertainty could be derived.

#### 3.2.1 Source/cause-identification confidence

**Figure 5:**
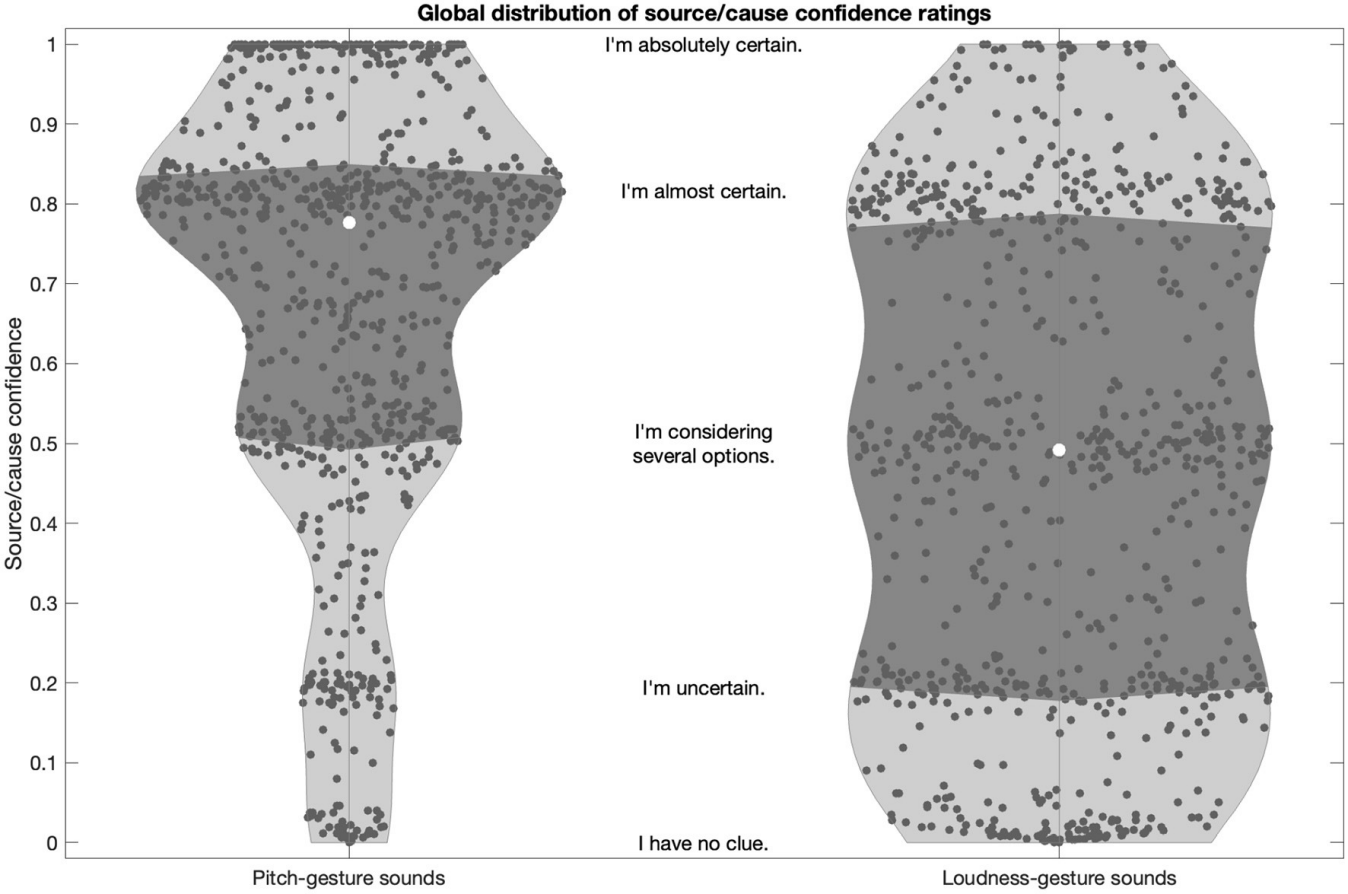
Global distribution of source/cause confidence ratings for pitch- and loudness-gesture sounds (left and right panels, respectively). White circles represent medians; darker shaded areas delimit the interquartile range. The textual descriptions represent the verbal labels of the rating scales in their approximate scale locations.

Figure 5 presents the global distributions of source/cause confidence ratings for pitch- and loudness-gesture sounds. For both sets of sounds, clustering of data points around the verbal anchors is evident, which suggests that the rating scale was mostly used as a categorical, ordinal scale, as opposed to a continuous, interval scale. This was considered in subsequent correlation analyses.

Source/cause confidence for pitch-gesture sounds was generally high (left panel), with half of the population falling between the verbal anchors “I'm almost certain” and “I'm considering several options”, with the median falling closer to the former. By contrast, source/cause confidence was markedly more variable across loudness-gesture sounds (right panel), with a nearly uniform spread over the entire scale range. Half of the ratings fell between the anchors “I'm almost certain” and “I'm uncertain”, with the median falling in the middle of the scale range. In sum, participants were more confident in identifying the source/cause in pitch- than in loudness-gesture sounds.

**Figure 6:**
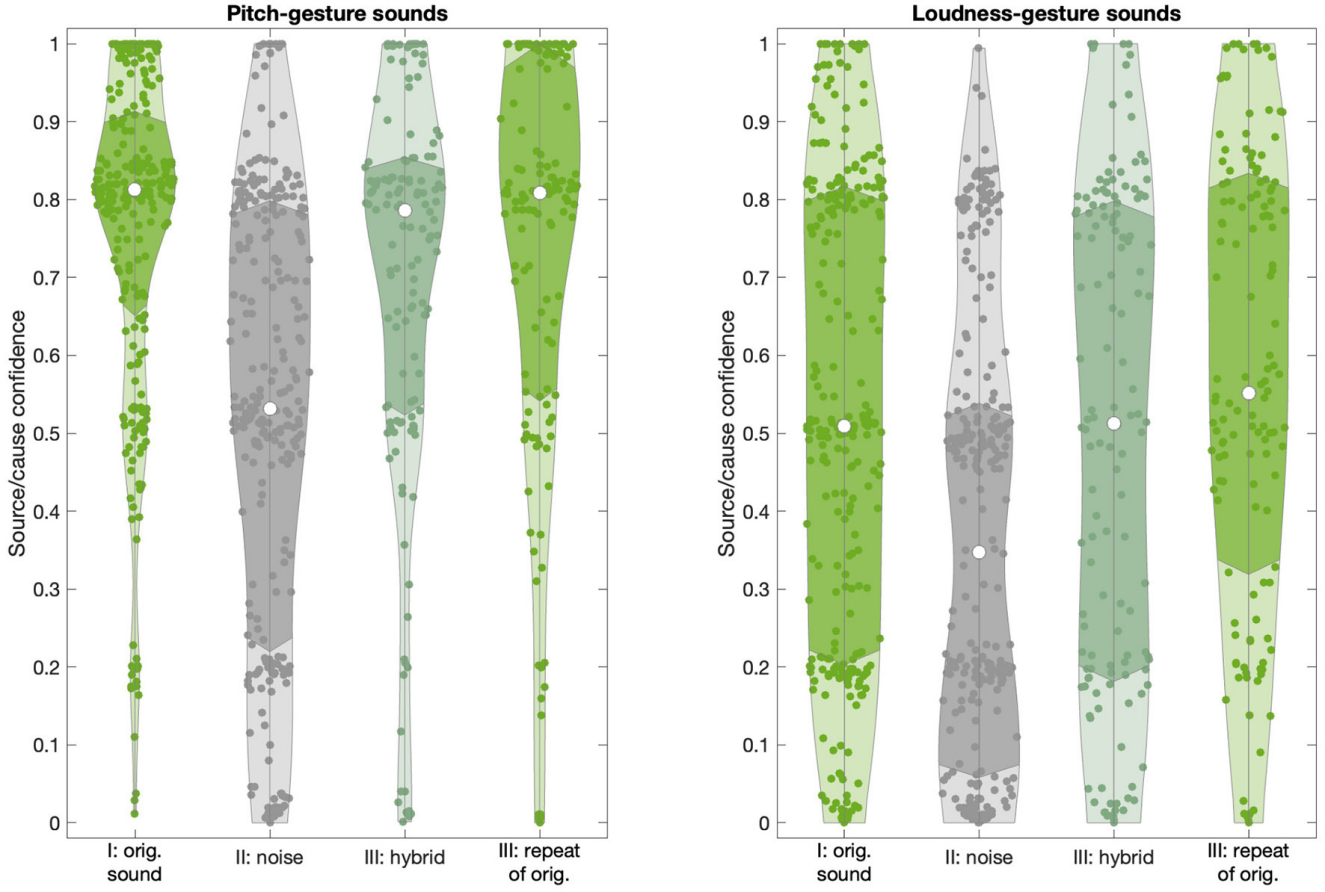
Distribution of source/cause confidence ratings across sound types (*original, noise-based, hybrid*) and presentation blocks (I, II, III) for pitch- and loudness-gesture sounds (left and right panels, respectively). White circles represent medians; darker shaded areas delimit the interquartile range.

Figure 6 reveals how source/cause confidence ratings varied as a function of sound type (*original, noise-based, hybrid*) and presentation blocks (I, II, III). For pitch-gesture sounds (left panel), given the attempted removal of source/cause identity cues, the *noise-based* sounds yielded a spread of rating values that stretched across the entire scale range. By contrast, *original* and *hybrid* sounds, for which identity cues were available, exhibit medians around the verbal anchor “I'm almost certain”, with more than 75% of ratings falling in the upper half of the scale range. Given the non-normal distributions, a Kruskal-Wallis test confirmed the difference in ratings between *noise-based* and *original*/*hybrid* sounds (χ^2^(3) = 117.1, *p* < 0.01).

For loudness-gesture sounds, the *noise-based* sounds yielded a positively skewed distribution, with 75% of ratings falling in the lower half of the scale range, reflecting low confidence. The distributions for *original* and *hybrid* sounds were wider than those for pitch-gesture sounds and centralized to the scale range and yielded medians around the verbal anchor “I'm considering several options”. Although less pronounced than for pitch-gesture sounds, the difference between *noise-based* and *original*/*hybrid* loudness-gesture sounds was significant (χ^2^(3) = 59.9, *p* < 0.01).

#### 3.2.2 Verbal description of source/cause

Participants provided verbal descriptions for the source/cause identity through two free-text fields for *material* and *action*. Due to the entry of verbal descriptions being optional, verbal descriptions were not always available, with the median proportion of available *material* and *action* entries across pitch-gesture sounds being 100% and 94%, respectively, while loudness-gesture sounds amounted to lower median proportions of 88% and 88%, respectively.

Given the unrestricted use of the text fields, entries required some manual editing and correction to address the following aims:

- Only nouns and qualifying adjectives were retained for *material*; only verbs and their particles were retained for *action*.
- Any connecting words such as prepositions, quantifiers, articles were removed.
- Multiple nouns and verbs per entry were accepted and considered as separate data. Similarly, verbs and particles were treated as separate word entries, which allowed similarities to be quantified more accurately, e.g., by treating “switch”, “turn”, “on”, and “off” separately.
- Verbs were transformed to their infinitive form (omitting the preceding particle “to”). This could involve participles and inversion of negations, e.g., “disengaged” adapted to “engage”. In four cases, the action required an object for its meaning to become clear, where either an object, as in “cut (grass)”, “lose (air)”, “give (gas)”, or a verb, as in “(cause) turbulence”, was added.
- Orthographical errors or deviations were corrected.

For each pitch/loudness-gesture sound, two independent lists of descriptions for *material* and *action* were compiled. Notably, not every participant provided data for each sound, while it was also possible for participants to contribute more than one word for *material* or *action* per sound. The word lists contained medians of 43 *material* and 37.5 *action* items for pitch-gesture sounds and 32 *material* and 27.5 *action* items for loudness-gesture sounds.

**Figure 7:**
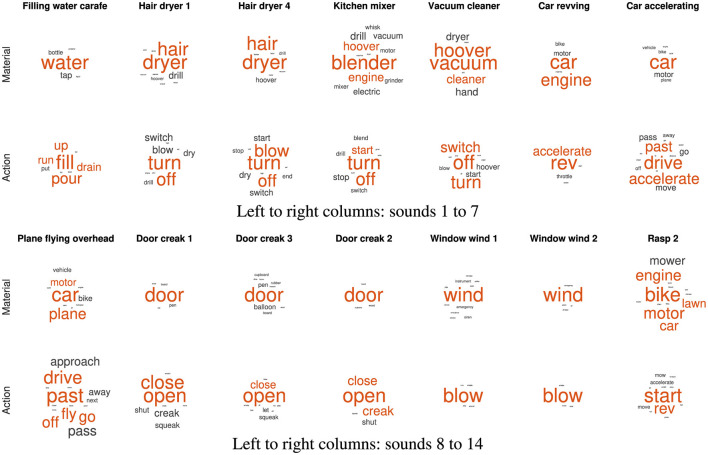
Word clouds for the 14 pitch-gesture *original* sounds based on verbal descriptions regarding *material* (upper rows) and *action* (lower rows). Font size corresponds to frequency of occurrence; orange color emphasizes the most frequent words employed.

**Figure 8:**
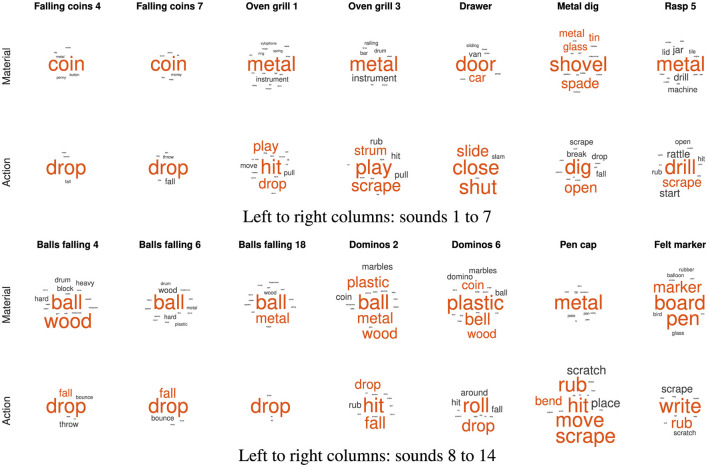
Word clouds for the 14 loudness-gesture *original* sounds based on verbal descriptions regarding *material* (upper rows) and *action* (lower rows). Font size corresponds to frequency of occurrence; orange color emphasizes the most frequent words employed.

The qualitative analysis of the word lists per sound considers all employed words and their relative frequencies of occurrence. As shown in Figures 7, 8 for pitch- and loudness-gesture sounds, respectively, lists for *material* (top row) and *action* (bottom row) served as the source to generate word clouds in MATLAB. Larger font size represents words' higher frequency of occurrence; the orange color emphasizes the most frequently employed words, whereas the spatial position of words carries no significance.

Figure 7 displays word clouds for the 14 pitch-gesture sounds (two sets of seven columns). Compared to the sounds' descriptions in Table 1, the most frequently employed words often match the actual materials and actions of the original sounds. Participants seemed able to precisely identify the sources and causes from the sounds, most notably for blowing wind, opening doors, filling water, and the operation of a hair dryer and a vacuum cleaner. Some ambiguity concerning the source concerned the airplane, the kitchen mixer, and the rasp, while the greatest variety concerning action descriptions applied to the airplane and the car.

Figure 8 contains word clouds for the 14 loudness-gesture sounds (two sets of seven columns). Verbal descriptions for loudness-gesture sounds exhibited a wider word palette than those for pitch-gesture sounds. Compared to the sounds' descriptions in Table 2, only about half of verbal descriptions matched the actual underlying sources. Notably, those concerned common objects like coins, balls, shovel, and marker pen. Descriptions for the remaining sounds did not identify the objects, but they appeared to identify probable physical materials involved, e.g., metal, plastic, wood. Concerning causes/actions, only falling or dropping coins and balls resulted in clear agreement, whereas the remaining sounds' descriptions employed a more diverse vocabulary.

The diversity of employed verbal descriptions can be quantified as the causal uncertainty score *H*_*cu*_. In the context of environmental sounds, this entropy measure has been used to quantify single noun-plus-verb descriptions (Ballas, 1993) or, when considering joint entropy, pairs of separate words for object and action (Lemaitre et al., 2010). Although the latter case likens the current distinction between materials and actions, it cannot be implemented in the same way due to the word lists for material and action here being independent and thus not exhibiting paired links. Instead, two separate causal uncertainty scores were computed for *material* and *action*, which also allows their independent analysis.

The same word lists for individual sounds that formed the word clouds in Figures 7, 8 served as basis for the causal uncertainty scores *H*_*cu*_. Similar to past uses of *H*_*cu*_ (Ballas, 1993; Lemaitre et al., 2010), three human evaluators independently sorted word-list items (for individual sounds) into groups based on semantic equivalence or distinction. For instance, actions like “pour” and “fill” or “turn” and “switch” could be deemed semantically equivalent; likewise, objects/materials like “hoover” and “vacuum” or “water” and “liquid” could be grouped together. The evaluators comprised an expert in sound-related semantics, a native-English linguist, and the author. The median number of groups (semantic categories) assigned by three evaluators were 7.5, 5, and 8.5 for *materials* and 10, 4.5, and 11 for *actions*, respectively.


(1) $$\begin{array}{c} Hcu_{i}=-\overset{n}{\underset{j}{\sum }}p_{ij}log_{2}\left(p_{ij}\right) \end{array}$$

Equation 1 describes the computation of the causal uncertainty score *Hcu*_*i*_ for the sound *i*. For a semantic category *j*, the proportion *p*_*ij*_ of its frequency of occurrence over the total number of list items is evaluated and weighted by its logarithmic transformation. This is conducted for all *n* semantic categories used to describe sound *i* and aggregated to a single uncertainty score. If all list items concern the same semantic category, *H*_*cu*_ = 0, whereas the uncertainty score increases with greater semantic diversity of items. The reliability of *Hcu*_*i*_ values between evaluators was high, *r*_1&2_(54) =.88, *r*_1&3_(54) =.95, *r*_2&3_(54) =.86, *p* < .0001, and compares to previous uses of *Hcu* (Ballas, 1993; Lemaitre et al., 2010). Given this reliability, the median *Hcu*_*i*_ was used in subsequent analysis.

**Figure 9:**
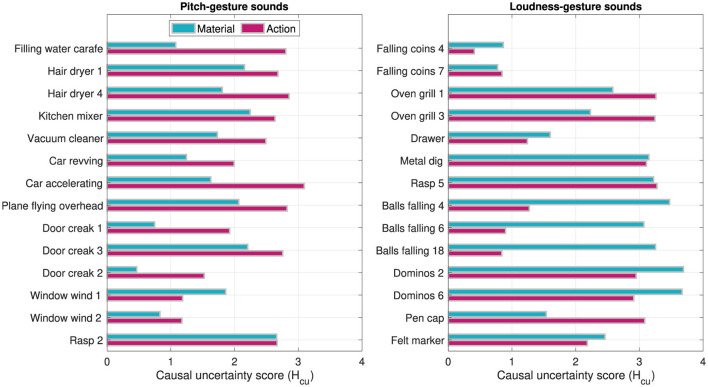
Causal uncertainty scores *Hcu* computed on single words employed in describing the *material* and *action* of 14 pitch-gesture- and 14 loudness-gesture sounds (left and right panels, respectively).

Figure 9 shows the computed causal uncertainty scores *Hcu* for *material* and *action* across the individual sounds. Whereas *Hcu* for *material* was on aggregate higher in loudness- than in pitch-gesture sounds, Wilcoxon rank-sum test: *z* = −2.41, *p* =.016, no such difference was found for *action*, *z* = −.25, *p* = 0.800. Comparing *Hcu* scores in Figure 9 to the word clouds in Figures 7, 8 exhibits some commonalities. For instance, the pitch-gesture sounds *Window wind 2* and *Rasp 2* illustrate the consistent vs. diverse use of verbal labels, respectively; an analogous contrast applies to the loudness-gesture sounds *Falling coins 4* and *Dominos 6*. It should be noted, however, that both representations are not equivalent, as the word clouds reflect lexical diversity, whereas the causal uncertainty scores express semantic diversity.

#### 3.2.3 Overall source/cause evaluation

**Figure 10:**
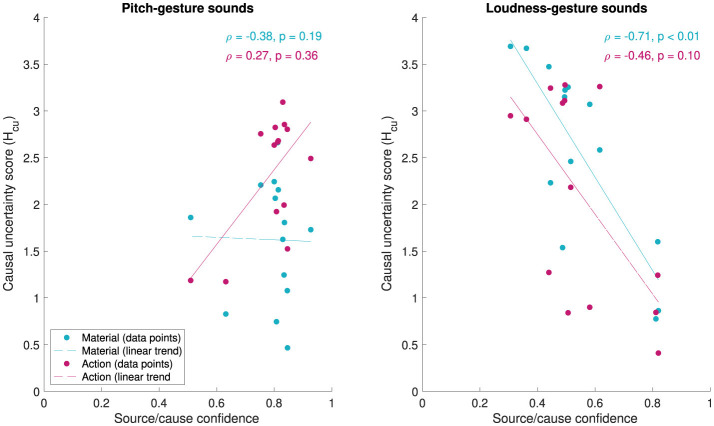
Median source/cause confidence ratings (x-axis) and causal uncertainty scores *Hcu* (y-axis) for 14 pitch-gesture- and 14 loudness-gesture sounds (left and right panels, respectively). *Hcu* concerns verbal descriptions for *material* and *action*. Spearman's ρ represents non-linear, rank correlation, while the linear trend lines are intended for illustration only.

Finally, Figure 10 relates the numerical measures for identification confidence and verbal descriptions of the source/cause to one another. The scatter plots visualize how median source/cause confidence ratings map onto causal uncertainty scores *Hcu* for *material* and *action* across the different *original* sounds. For pitch-gesture sounds (left panel), a narrower range of source/cause confidence values maps onto a wider spread of causal uncertainty values, with no clear correlation (Spearman's ρ) between the two evident (*material*: ρ = −.38, *p* =.19, *action*: ρ =.27, *p* =.36). For loudness-gesture sounds, by contrast, values across both scales are dispersed more, here yielding negatively correlated trends for both verbal attributes, although only the one for *material* is significant (*material*: ρ = −.71, *p* < .01, *action*: ρ = −.46, *p* =.10).

### 3.3 Relationship between gesture identification and source/cause evaluation

**Figure 11:**
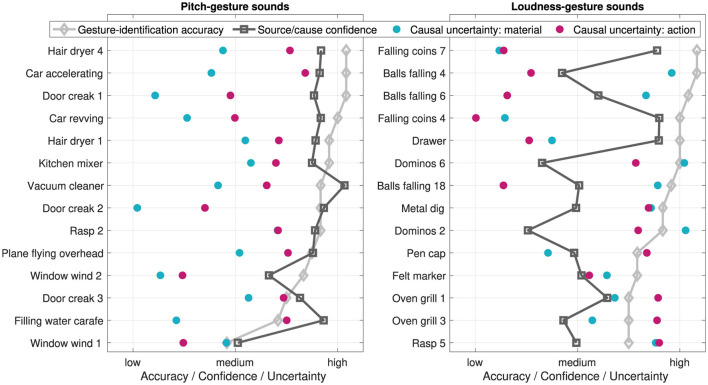
Gesture-identification *accuracy*, source/cause-identification *confidence*, and causal *uncertainty* for *original* environmental sounds across 14 pitch and 14 loudness gestures (left and right panels, respectively), ordered from highest to lowest *accuracy*. Data is normalized to the respective scale ranges for comparability.

Figure 11 provides an overview how gesture-identification *accuracy*, source/cause-identification *confidence*, and causal *uncertainty* in verbally describing materials and actions relate to each other across the 14 pitch-gesture and 14 loudness-gesture sounds (left and right panels, respectively). In each pitch/loudness group, the sounds are ordered from highest to lowest gesture-identification accuracy to aid interpretation.

For pitch-gesture sounds, a visual inspection suggests that as gesture-identification accuracy decreases so does the source/cause-identification confidence. This did not amount to a clear correlation, however (ρ =.35, *p* =.22). Similarly, also causal uncertainty appeared largely independent of gesture-identification accuracy (*material*: ρ = −.16, *p* =.60, *action*: ρ =.32, *p* =.27). Loudness-gesture sounds exhibited hardly any correlation between gesture-identification accuracy and source/cause-identification confidence (ρ =.18, *p* =.53) and causal uncertainty for *material* (ρ = −.06, *p* =.84). A notable exception is found in the clear negative correlation between gesture-identification accuracy and causal uncertainty for *action* (ρ = −.75, *p < .01*).

## 4 Discussion

The reported study sought to determine to what extent listeners with no specialized training can attend to the time course of sound qualities like pitch or loudness, here termed sound gestures, while also being asked to evaluate the underlying source or cause of the sound. For a given environmental sound, listeners were asked to identify sound gestures through crossmodal, visual analogues, to rate their confidence in identifying the source/cause, and to provide descriptions for the source (material) and cause (action).

### 4.1 Crossmodal features and sound gestures

Results suggest that participants were able to use the crossmodal-matching task to identify the underlying gestures, which confirms the utility of the used two-dimensional visual interface also in the context of environmental sounds. Indeed, in western cultures horizontal space can intuitively be used to represent time (Athanasopoulos and Moran, 2013; Küssner and Leech-Wilkinson, 2014; Lacey et al., 2020; Lembke, 2023). For pitch gestures, the use of the vertical dimension reflects the ubiquitous pitch-to-elevation correspondence (e.g., Walker, 1987; Prince et al., 2009; Spence, 2011; Athanasopoulos and Moran, 2013; Küssner and Leech-Wilkinson, 2014; Lacey et al., 2020; Lembke, 2023; Getz, 2023), while participants displayed no obvious issues mapping loudness onto the vertical dimension (Eitan et al., 2008; Küssner and Leech-Wilkinson, 2014; Bruzzi et al., 2017; Lembke, 2023). Overall, the crossmodal interface appeared to serve as a reliable tool to measure the identification of sound gestures.

Listeners seemed better at identifying pitch and loudness gestures based on features related to macro contour, whereas listeners appeared to not consider (or perceive) more detailed features to the same extent (compare *relaxed* vs *strict* classification, respectively, in Figure 2). As this investigation sought to describe listeners' general ability of sound-gesture identification and to consider its utility to applications in crossmodal interfaces, e.g., description of sound gestures via visual analogues, we chose to limit the subsequent analysis and discussion of results to the macro representation. It can be argued that an identification accuracy of 80% or more would be needed to be of practical value in such crossmodal applications.

Although sound-gesture identification based on macro contour seemed more reliable here, under different experimental constraints and an exclusive focus on gestural features, listeners can distinguish finer-grained differences across varying degrees of pitch-gesture shape (Lembke, 2023), resembling the different degrees of curvature found across the pitch gestures depicted in Table 3. Given the identification accuracy of 81–83%, it can be concluded that macro features (e.g., *rise, plateau, fall*) carry greater informational weight than fine-grained, shape-related differences, which were identified with only 50–55% accuracy, in both cases reliably above chance. The greater reliance on macro features compares to findings observed for the auditory comparison of pitch contours from speech prosody (Hermes, 1998). Furthermore, insights from conveying emotions through speech prosody also point to a greater importance of macro features, while fine-grained features are still acknowledged as being relevant (Mozziconacci and Hermes, 1999; Mozziconacci, 2001). Finally, as linear correlation is known to predict the similarity between melodic pitch contours (Prince et al., 2009), similarity in macro contour can be expected to influence the magnitude of correlations to a greater degree than finer details of the contour.

Based on the macro contour (*relaxed* classification), identification accuracy did not substantially depend on whether gestures were embedded in the original environmental sounds or emphasized in manipulated variants (see Figure 3A). For pitch gestures, correct identifications increased by only 6% when emphasizing gestures through noise-based frequency trajectories, which suggests that some aspect related to the acoustic complexity of the original sounds may have masked salient features that convey pitch gestures to a limited degree. Such masking did not prevent accuracies above 80% to be achieved for the original sounds regardless. For loudness gestures, no difference in identification could be observed, which suggests that the temporal variation of loudness underlying the gestures was no less salient in the original environmental sounds.

Whereas the repeated presentation of the same gesture in a separate trial did not seem to affect identification accuracy (see Figure 3B), accuracy clearly varied across the set of 28 environmental sounds (see Figure 4). Pitch gestures exhibited the widest range of accuracies, with only two sounds performing around chance level, *Window wind 1* and *Filling water carafe*. Although correlational analyses involving possible explanatory acoustic and categorical variables were explored, they did not provide meaningful insights. Instead, sounds yielding lower identification accuracy will at least be considered qualitatively.

The sound *Window wind 1* involves air flow that generates a clear, tonal “whistling” trajectory. Notably, as the whistling swells in amplitude, the inharmonically related second partial tone becomes briefly more prominent than the fundamental tone, forming an interval of about a tritone (half-octave). By contrast, the visual analogue was modeled by following only the fundamental tone. As this fails to account for the more drastic inflection of pitch, it may therefore not have sufficiently emphasized the perceived auditory *rise* by instead conveying a *plateau*. This inaccuracy in the visual analogue may have rendered the “correct” identification impossible and thus explain the performance at chance level, which finds further support in the markedly higher identification accuracy obtained for the *noise-based*, isolated sound gesture (see corresponding letter N in Figure 4), which matched the visual analogue.

The marginally reliable identification of the sound *Filling water carafe* may be related to a lacking salience of the pitch gesture (see Lemaitre et al., 2017, for comparable example). Unlike other environmental sounds that exhibit tonal components, the water filling concerns a granular, noise-like sound source. Filling of the carafe results in the continuous shortening of the resonating air-column inside it. With the water source being modified by the dynamic change in column length, the resulting sound resembles a more timbre-related, filtered brightness trajectory that is less salient than pitch conveyed through tonal components. Also this interpretation is further supported by the same gesture's more salient occurrence in the *noise-based* version that achieved considerably higher identification accuracy (see letter N).

In some cases, there may also be multiple ambiguous gesture cues. Whereas for the sound *Plane flying overhead* the plane motor's tonal components outline the Doppler frequency shift typical for approximating and receding sound sources, as depicted by the corresponding visual analogue (see Table 3), a similar airplane-flyover sound (Freesound ID 211870), which was excluded from consideration for the experiment, exhibits a separate, clearly audible flanger-effect trajectory resulting from continual changes to the delay(s) between the plane's direct and reflected sound paths. For pitch gestures, therefore, insufficient feature salience or clarity may impose significant constraints on the identifiability of gestures in environmental sounds, where already for synthetic frequency contours the “clarity of delineation” has been argued to hold special importance (Walker, 1987, p. 501).

Among the sounds involving loudness gestures, only for the sound *Dominos 6* was a difference in identification performance between *original* and *noise-based* versions significant. Contrary to the aim for noise-based reductions to emphasize sound gestures, listeners exhibited a markedly lower identification accuracy for this sound type. As the low-pass filter used to extract the loudness gesture introduced a degree of temporal smoothing, this could have in turn reduced the clarity of the impacts between wood pieces. Although no similar differences emerged for other iterative impulsive sounds, such as dominos or oven grill, this single finding could still point to the auditory salience for loudness gestures relying on a sufficiently fine resolution of temporal features.

### 4.2 Source-related features

While the experiment sought to measure listeners' ability to identify sound gestures occurring in environmental sounds, it intentionally engaged listeners with the concurrent task of evaluating the sounds' source or cause. Participants' confidence ratings provided an estimate of the identifiability of the source/cause, where the markedly lower confidence observed for the noise-based reductions (see Figure 6) suggests that the chosen sound manipulations succeeded in obscuring the original environmental sounds' identity. The second means of engagement required participants to verbally describe the material (object) and action (cause), the two most common categorization attributes for environmental sounds (Guastavino, 2018; Giordano et al., 2022). The manipulated reductions also yielded ambiguity in the verbal descriptions. Noise-based pitch gestures were described in terms of plausible causes like “wind”, while the most frequent labels for loudness gestures concerned the unspecific terms “object” and “thing” (corresponding word clouds omitted for space reasons). The main utility of these source/cause measures, however, was to assess their relevance to the perception of the original sounds.

Concerning the original environmental sounds, participants were overall more confident in determining the source/cause underlying the 14 sounds containing pitch gestures compared to the 14 sounds related to loudness gestures (see Figures 5, 11). This marked difference could be related to the former involving objects and actions from familiar domestic or urban contexts, e.g., operating hair dryers, creaking doors, whereas most of the loudness-gesture sounds involved materials and actions that were overall more ambiguous, e.g., sequence of dominos falling, strumming across oven grill. Also for loudness-gesture sounds, familiar contexts such as falling coins or closing of drawers yielded high confidence, which agrees with a previous report of sound familiarity and identifiability correlating strongly (Ballas, 1993). As for gesture identification, the repeated presentation of a sound did not affect confidence ratings to a large degree (see Blocks I vs III in Figure 6).

Next to the confidence ratings, participants employed verbal descriptions concerning *materials* (objects) and *actions* (causes) underlying the environmental sounds. These attributes have been similarly relevant to previous studies (e.g., Ballas, 1993; Lemaitre et al., 2010; Lemaitre and Heller, 2013; Guastavino, 2018; Giordano et al., 2022). Although the descriptions were not used to quantify correct identifications, the most frequently employed descriptions did mostly match the actual underlying sources and causes (compare Figures 7, 8 with Tables 1, 2, respectively).

The categorization of verbal descriptions based on semantic equivalence allowed the consideration of the previously employed causal-uncertainty score *Hcu* (Ballas, 1993; Lemaitre et al., 2010), which were separately determined from descriptions of *materials* and *actions*. Mirroring a similar trend across confidence ratings, *Hcu* for *material* was generally lower in pitch- than in loudness-gesture sounds, which signifies greater semantic agreement across participants verbal descriptions for the former group of environmental sounds. As no comparable difference was observed for *Hcu* related to *actions*, this may relate to the determination of underlying *materials* requiring greater cognitive effort (Houix et al., 2012; Lemaitre and Heller, 2012; Lemaitre et al., 2018), which therefore could have amplified differences between the pitch- and loudness-gesture sounds.

With regard to the relationship between measures related to source/cause identification, lacking confidence in the ability to identify the source/cause appeared to partly correlate with greater semantic uncertainty in verbal descriptions for the loudness- but not the pitch-gesture sounds (see Figure 10). Similar negative correlations have been reported previously (Ballas, 1993; Lemaitre et al., 2010). However, the findings of Lemaitre et al. (2010) also included pairings of high confidence and high uncertainty, a trend that can be observed in the here investigated pitch-gesture sounds for *actions*. In a related context, greater source identifiability/confidence has been found to correlate with increased identification accuracy (Ballas, 1993; Dickerson et al., 2018), which here can be supported qualitatively for only loudness-, e.g., *Falling coins 7* vs *Dominos 2*, but not pitch-gesture sounds, e.g., *Hair dryer 4* vs *Window wind 1* (compare Figure 11 with word clouds in Figures 7, 8).

### 4.3 Relationship between source-related and crossmodal features

In order to address all experimental tasks, participants had to attend to auditory features that conveyed both pitch/loudness gestures and source/cause identity. Given the need to attend to a wider, more varied set of relevant auditory features, potential interactions in addressing the tasks of gesture identification, rating of source/cause confidence, and verbal descriptions could have occurred. Overall, the relationship between the measures (see Figure 11) provides no clear evidence for there to be an interaction one way or another. Participants' performance in the experimental task of gesture identification appeared largely independent of their performance in both tasks related to source/cause identification.

The only exception concerned the relationship between high gesture-identification accuracy and the low causal uncertainty with regard to the consistent attribution of “falling” or “dropping” actions related to balls and coins (among loudness-gesture sounds), whereas greater causal uncertainty concerning oven grill and rasp occurred alongside poorer gesture-identification performance. These pairings appeared to mainly drive the observed negative correlation between causal uncertainty (*H*_*cu*_) and gesture-identification accuracy. Differences in the temporal complexity of the sounds could explain why both measures varied this way. For instance, falling coins or balls provide rhythmically more distinct and simple patterns compared to the rapid, iterative, and complex patterns of the rasp and oven-grill sounds (see Table 4), in which case less temporal complexity could have favored both gesture-identification accuracy and low causal uncertainty.

The independence between evaluations of sound-gesture and source/cause properties suggests that participants were able to attend to all tasks without interference. This apparent task independence stands in contrast to previous findings that showed how sounds with greater identifiability/confidence favored their evaluation to focus on source/cause properties, while greater causal uncertainty favored evaluation based on acoustic properties (Lemaitre et al., 2010), with the latter properties here relating to pitch/loudness variation. Notably, Lemaitre et al. (2010) asked listeners to freely sort sounds based on their inherent similarity, which allowed categorizations to reflect either causal or acoustic bases. By contrast, listeners in the current study were tasked with engaging with both acoustic (pitch/loudness) and causal properties. While it therefore can be expected for source/cause properties to affect the degree to which listeners spontaneously engage with acoustic or causal properties, the findings from this study complement this knowledge by showing listeners' ability to engage with both properties alongside each other without apparent interaction.

On a theoretical level, the experimental tasks required participants to engage with both *everyday* and *musical* modes of listening (Gaver, 1993), both of which they seemed capable engaging with concurrently. As participants reliably identified sound gestures regardless of whether the source/cause identity was obscured or not, both modes of listening did not appear to interfere with each other. It should be noted that observed differences in gesture-identification accuracy between manipulated and original sounds were small and more likely caused by differences in feature salience than modes of listening. Although Smalley (1997) argued that sounds with a clear *bond* to their physical source or cause detract listeners from evaluating those intrinsic features that convey sound gestures, e.g., pitch/loudness, the findings obtained here paint a different picture, in that listeners evaluation of intrinsic features seemed largely unaffected by whether the extrinsic *source bond* was available or absent, which resembles reports of direct evaluations of timbral brightness in sounds of musical instruments remaining independent from underlying source/cause categories (Saitis and Siedenburg, 2020).

Given that listeners performed well across all experimental tasks without interference between them, future studies could explore whether pitch or loudness gestures occurring in environmental sounds could in fact contribute to source/cause identification. In other words, could the temporal variation of acoustic qualities help establish the causal “narrative” underlying the environmental sounds? Many environmental sounds studied here concerned compound events, as they involved a sequence of basic-level events (Gaver, 1993). As these basic events could also relate to individual stages of gestural macro contour, e.g., the contour *rise, plateau, fall* signifying “turn on”, “remain on”, “turn off”, they could underpin the important role temporal patterning plays in the perception of environmental sounds (Gaver, 1993; Houix et al., 2012).

In a similar vein, the simple, distinct temporal patterns of balls or coins falling and bouncing conveyed though loudness gestures could have already conveyed the underlying actions. Indeed, features related to only the temporal amplitude envelope can convey the underlying actions or causal events in certain environmental sounds, such as for bouncing objects (Warren and Verbrugge, 1984), dropping and striking excitations (Hjortkjær and McAdams, 2016), and characteristic impulsive patterns of helicopters, ping-pong playing or hand clapping (Gygi et al., 2004; Shafiro, 2008).

With the recognition of sound sources shown to depend on the magnitude of sound qualities (e.g., loudness, Susini et al., 2019; Traer et al., 2021), the question remains to what extent and in what way it might similarly be influenced by dynamic changes in pitch, loudness or other sound qualities. In dynamic everyday environments, humans experience the common scenario of largely correlated multimodal cues. Due to frequent changes in spatial configuration and orientation, availability of visual cues is more likely to be fragmented while auditory cues remain available and may thus provide a sense of continuity through crossmodal complementarity. Such examples could serve as scenarios to investigate how crossmodal correspondences relate to human adaptation in environments (Parise, 2016).

Associations between sounds and human manual actions furthermore offer a variety of potential benefits, for instance, with embodied motion cues facilitating the learning of sound-to-action associations (Navolio et al., 2016) or congruence of sound source and object material influencing manual actions (Castiello et al., 2010). In this context, the question arises to what degree some of the environmental sounds studied here could have benefitted from human motions involved in their sound generation and whether the motions could have aided establishing the crossmodal link to sound gestures. For loudness gestures, the sounds involving balls or coins exhibit clear parallels between their dropping and bouncing motions and loudness contours, which could thus have facilitated their association. By contrast, the direct manual handling of pen cap and felt marker did not result in their respective sounds achieving better loudness-gesture identification compared to those related to only a single tipping motion triggering a sequence of falling dominos.

Among the actions underlying the pitch gestures, the most immediate manual handling concerned the filling of the carafe, with the gradually rotating pouring gesture going hand in hand with the rising pitch gesture. As discussed earlier, however, this did not seem to aid the reliable identification of the latter. More generally, manual handling or operation can occur at varying levels of agency. Although the pitch gestures in the operation of hair dryer, kitchen mixer, and vacuum cleaner were well identified, the binary switching motions involved in their operation hardly resemble the continuous pitch gestures underlying them, while they still demarcate the individual stages of the gestures' macro contour. Overall, whereas the selected 28 environmental sounds helped to pursue the exploratory aim of studying the identification of sound gestures, the selection does not constitute a representative sample of how human agency or perspectives could play a role. To confirm the latter would require a larger sample of sounds and one that ensures balance across categories such as ego- and exocentric frames of reference (Navolio et al., 2016), living and nonliving or action and nonaction sounds (Giordano et al., 2010).

In future studies, listening expertise should be considered as a factor, given that it affects the perception of both environmental sounds and sound gestures. Expert listeners display a greater tendency to evaluate properties related to sound qualities over sound source/cause (Lemaitre et al., 2010), while also the perception of sound gestures or pitch contours becomes more accurate with greater listening expertise (Balch and Muscatelli, 1986; Walker, 1987; Prince et al., 2009; Küssner et al., 2014; Küssner and Leech-Wilkinson, 2014). The current study deliberately recruited participants with limited expertise to potentially augment the effect of *source bonding* (Smalley, 1997), that is, clear source/cause identity interfering with the evaluation of sound gestures. With our findings showing no such negative impact, it remains to be investigated whether greater expertise could improve identification accuracy based on fine-grained, shape-related gesture features (*strict* classification), in which context the differences between macro and fine-grained contour could be varied parametrically.

In conclusion, even with limited expertise, listeners can reliably identify sound gestures occurring in environmental sounds based on features related to the gestures' macro contour. Listeners can achieve this while concurrently evaluating properties related to the sound source and cause, which suggests that listeners can engage with both tasks independently. Only for pitch-based gestures does the identification accuracy notably vary with auditory salience and acoustic complexity of individual environmental sounds, whereas enhancing salience in loudness-based gestures makes little difference. The reliable, intuitive crossmodal identification of sound gestures and previous reports of cognitive advantages for visual-to-auditory interfaces (Balch and Muscatelli, 1986; Prince et al., 2009) promote the utility of such interfaces, where they could underpin query-by-example searches for sounds based on visual depictions in various applications, e.g., sound design or synthesis (e.g., Miranda et al., 2000), emotion recognition in speech (Williams and Stevens, 1972; Mozziconacci, 2001). Whether the crossmodal associations evoked in environmental sounds extend to carrying the ecologically relevant role of conveying causal narratives remains to be explored.

## Data Availability

The original contributions presented in the study are included in the article/Supplementary material, further inquiries can be directed to the corresponding author.

## Footnotes

^1^URL: https://freesound.org/.

^2^URL: https://soundcloud.com.
